# Long-Range Interocular Suppression in Adults with Strabismic Amblyopia: A Pilot fMRI Study

**DOI:** 10.3390/vision3010002

**Published:** 2019-01-08

**Authors:** Benjamin Thompson, Goro Maehara, Erin Goddard, Reza Farivar, Behzad Mansouri, Robert F. Hess

**Affiliations:** 1 School of Optometry and Vision Science, University of Waterloo, Waterloo, ON N2L 3G1, Canada; 2 McGill Vision Research, Department of Ophthalmology and Visual Sciences, McGill University, Montreal, QC H4A 3S5, Canada; 3 School of Optometry and Vision Science, University of Auckland, Auckland 1142, New Zealand; 4 Department of Human Sciences, Kanagawa University, Yokohama 221-8686, Japan; 5 Department of Ophthalmology, University of Manitoba, Winnipeg, MB R3T 2N2, Canada

**Keywords:** visual development, binocular vision, functional magnetic resonance imaging, strabismus, visual processing, primary visual cortex

## Abstract

Interocular suppression plays an important role in the visual deficits experienced by individuals with amblyopia. Most neurophysiological and functional MRI studies of suppression in amblyopia have used dichoptic stimuli that overlap within the visual field. However, suppression of the amblyopic eye also occurs when the dichoptic stimuli do not overlap, a phenomenon we refer to as long-range suppression. We used functional MRI to test the hypothesis that long-range suppression reduces neural activity in V1, V2 and V3 in adults with amblyopia, indicative of an early, active inhibition mechanism. Five adults with amblyopia and five controls viewed monocular and dichoptic quadrant stimuli during fMRI. Three of five participants with amblyopia experienced complete perceptual suppression of the quadrants presented to their amblyopic eye under dichoptic viewing. The blood oxygen level dependant (BOLD) responses within retinotopic regions corresponding to amblyopic and fellow eye stimuli were analyzed for response magnitude, time to peak, effective connectivity and stimulus classification. Dichoptic viewing slightly reduced the BOLD response magnitude in amblyopic eye retinotopic regions in V1 and reduced the time to peak response; however, the same effects were also present in the non-dominant eye of controls. Effective connectivity was unaffected by suppression, and the results of a classification analysis did not differ significantly between the control and amblyopia groups. Overall, we did not observe a neural signature of long-range amblyopic eye suppression in V1, V2 or V3 using functional MRI in this initial study. This type of suppression may involve higher level processing areas within the brain.

## 1. Introduction

Strabismus in infancy can result in chronic interocular suppression of the deviated eye and the development of strabismic amblyopia. Strabismic amblyopic eyes exhibit a wide range of visual deficits including excessive crowding [1,2,3], abnormal spatial summation [4] and impairments in visual acuity [5], contrast sensitivity [6] and global motion perception [7,8] (see [1,9] for reviews). In addition, strabismic amblyopia is accompanied by a loss of binocular visual functions such as stereopsis. In recent years, it has become apparent that both the monocular and binocular deficits caused by strabismic amblyopia are associated with the strength of interocular suppression [10,11,12,13,14]. In particular, stronger suppression is correlated with poorer amblyopic eye visual acuity and poorer stereoscopic depth perception [12,15,16,17].

Animal models have demonstrated that strabismic suppression involves GABA-mediated inhibition within cat V1 [18] and that strabismic amblyopia increases the extent of inhibitory interactions in primate V1 and V2 [19,20]. Recent primate studies have also indicated that suppression in amblyopia is associated with an altered balance of inhibition and excitation in V1 and V2 that results in a reduced inhibitory drive from the amblyopic eye to the fellow eye [21,22]. It is currently unclear whether suppression is caused by active suppression of amblyopic eye signals, weak and noisy inputs from the amblyopic eye to a normal interocular inhibition mechanism [23] or a combination of both.

Functional MRI studies of humans with strabismic amblyopia have primarily investigated cortical activation under monocular viewing conditions. However, a small number of fMRI studies have employed dichoptic viewing paradigms that have enabled a manipulation of suppression. Conner et al. [24] presented retinotopic mapping stimuli to the amblyopic eye while the fellow eye viewed mean luminance or was occluded. One of the four participants tested had strabismic amblyopia due to infantile esotropia and reported suppression of the amblyopic eye. This participant exhibited an attenuated blood oxygen level dependant (BOLD) response in V1 for the mean luminance relative to the occlusion condition, a difference that could be associated with the participant’s perceptual experience of amblyopic eye suppression. In addition, we have previously reported that interocular suppression causes a delayed and attenuated V1 BOLD response in participants with strabismic amblyopia [25]. This experiment involved presenting a stationary fractal noise pattern to the fellow eye while the amblyopic eye viewed briefly presented dynamic fractal noise patterns.

Both of the studies described above investigated interocular suppression using dichoptic stimuli that overlap within the visual field. However, interocular suppression can also be induced by dichoptic stimuli that do not overlap and therefore stimulate distinct populations of receptive fields within V1 and V2. In participants with normal binocular vision, both surround suppression and lateral inhibition occur for dichoptic stimuli [26,27]. Furthermore, in amblyopia, interocular suppression can be induced by dichoptic stimuli that are presented to separate quadrants of the visual field. We refer to this phenomenon as long-range suppression. For example, Maehara et al. [28] presented four gratings to participants with amblyopia, one in each quadrant of the visual field. When the gratings were split equally between the eyes, interocular suppression reduced the perceived luminance of the gratings presented to the amblyopic eye and in some cases caused complete suppression of the amblyopic eye gratings. A similar effect can be induced by splitting portions of an annulus stimulus between the eyes [15,17,29]. Moreover, the strength of suppression can be equivalent between overlapping and non-overlapping stimuli in some situations. For example, Huang et al. [30] showed that overlay and surround suppression for dichoptic contrast noise stimuli were of comparable strength in amblyopia.

Here, we used fMRI and the visual field quadrant stimulus design described by Maehara et al. [28] to investigate whether long-range suppression in strabismic amblyopia is evident within the primary and/or early extrastriate visual cortex. Our motivation was two-fold. First, it is currently unclear whether long-range suppression originates from binocular interactions in V1 and V2, as appears to be the case for overlapping stimuli [19,20,21,22,25,31]. Second, long-range suppression offers an attractive opportunity to study interocular suppression using fMRI, because suppressed and non-suppressed stimulus elements are restricted to unique retinotopic locations in primary and extrastriate visual areas. Our hypothesis was that suppression would attenuate and delay the BOLD response in V1 for stimuli presented to the amblyopic eye, as is the case for suppression induced by overlapping stimuli [25]. We tested this hypothesis using standard measures of BOLD response magnitude and time to peak response.

We also conducted two further analyses to assess the influence of long-range suppression on cortical activation in amblyopia. First, we investigated whether long-range suppression modulated feedforward or feedback effective connectivity between visual areas. Connectivity between and within visual areas is impaired in amblyopia both at rest [32,33,34,35] and during visual task performance [36,37]. For example, we have previously observed that effective connectivity is reduced between a range of visual areas including V1, V2 and V3 under monocular amblyopic eye vs. fellow eye viewing conditions [36]. As a whole, connectivity studies have indicated that amblyopia disrupts interactions within visual networks and also impairs the interaction between visual networks and networks involved in attention. Here, we tested whether long-range suppression of the amblyopic eye would further degrade effective connectivity between V1, V2 and V3 relative to monocular amblyopic eye viewing. We analyzed feedforward and feedback effective connectivity separately [36] based on the hypothesis that an early, active suppression mechanism would impair both feedforward and feedback connectivity, whereas a higher-level mechanism involving functions such as attention [37] would selectively impair feedback connectivity. Secondly, we tested whether a classification analysis would reveal a difference in the pattern of voxel-by-voxel BOLD activation within V1, V2 or V3 when long-range suppression was active vs. inactive. Classification analyses are able to detect subtle differences in the pattern of cortical activation that may not be evident in measures of BOLD response magnitude [38].

## 2. Methods

### 2.1. Participants

Five adults with strabismic amblyopia and five control observers with best-corrected logMAR visual acuity of 0.00 or better in each eye and normal Randot stereopsis (mean age 35 years, range 25–58; two participants wore refractive correction in the scanner) participated. One additional participant with strabismic amblyopia was excluded from the study because the participant fused the dichoptic stimuli during fMRI, which prevented localization of the retinotopic areas corresponding to stimulation of the amblyopic vs. the fellow eye (see below). Patient details are provided in Table 1. All patients had strabismus; one also had anisometropia. Amblyopia was defined as an interocular visual acuity difference of at least 0.2 logMAR with otherwise healthy eyes. Anisometropia was defined as an interocular subjective refractive error difference of 2.0 DS spherical equivalent. Visual acuity was measured using an ETDRS chart from 6 m. Stereopsis was measured using the Randot test according to the manufacturer’s instructions. Strabismus angle was measured using a synoptophore. Eye dominance in controls was determined using the Dorma sighting test. All participants provided full written informed consent. The study protocols conformed to the Declaration of Helsinki and were approved by the McGill University ethics review board.

### 2.2. Stimuli

**Figure 1:**
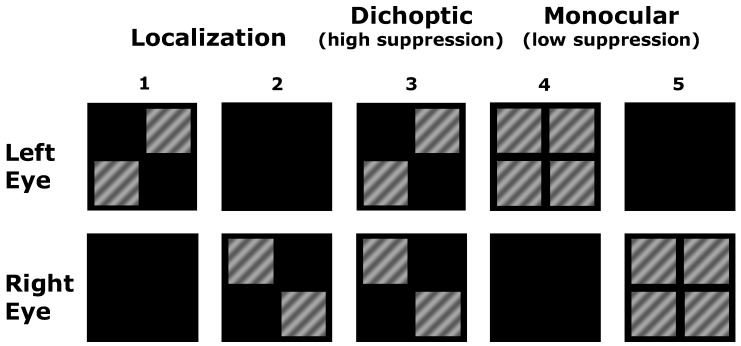
A schematic of the different stimulus configurations used during fMRI. The column numbers are used within the main text to refer to localization (1 and 2), dichoptic (3) and monocular (4 and 5) stimulus configurations.

Functional MRI stimuli were based on those used by Maehara et al. [28]. Participants fixated a mark at the center of the presentation screen. Square patches of sinusoidal grating (30% contrast, 0.5 cycles per degree (cpd), 7° width) were presented in two or more quadrants of the screen according to the monocular and dichoptic configurations depicted in Figure 1. Areas of the screen with no grating were dark. The two monocular configurations involving only two patches (Figure 1, Columns 1 and 2) were used to localize retinotopic regions of interest for left and right eye stimuli separately. The monocular configuration with four patches (Figure 1, Columns 4 and 5) and the dichoptic configuration (Figure 1, Column 3) were compared to assess the effect of suppression (dichoptic presentation) relative to no suppression (monocular presentation). Note that stimulation of the eye-specific quadrants was identical in the dichoptic and monocular conditions; the only difference between conditions was whether the remaining quadrants were presented to the other eye (dichoptic condition, high suppression) or to the same eye (monocular condition, low suppression).

Prisms were used to cancel manifest strabismus for participants GN, JG and AS (no participants habitually wore prismatic correction), and participants wore full optical correction in MR-compatible frames. During fMRI, participants performed a reaction time task whereby they pressed a button every time the fixation mark changed from a circle to a square. These changes were frequent and demanded sustained fixation. For the dichoptic stimulus configuration, the fixation mark was presented to the fellow fixing/dominant eye to ensure that it was visible and not subject to suppression. We did not present the fixation mark to both eyes because we wanted to maximize suppression of the amblyopic eye in the dichoptic condition. For monocular configurations, the fixation mark was presented to the eye receiving the stimuli. While participants performed the central task, grating stimuli were presented for 1500 ms with a 500-ms interstimulus interval. There were 8 stimuli per block and stimulus blocks were interleaved with blocks of blank fixation. Each block lasted 16 seconds, and each stimulus configuration was repeated 3 times per scanning run. Each participant completed a minimum of 5 scanning runs. Dichoptic stimuli were presented using an MR-compatible cross-polarized dual projection system that has been described in detail elsewhere [39]. The two projectors were linearly calibrated to ensure equal projection of contrast modulated stimuli and the mean luminance of the grating stimuli was also matched between the two projectors (46 ± 2 cd/m^2^ when measurements were made through the polarizers).

### 2.3. Psychophysical Measurements

Participants completed the dichoptic luminance matching procedure described by Mahara et al. [28]. Dichoptic pairs of gratings (2 × 2 ° per patch, 1 cpd, 25% contrast) were presented on a dark background. Reference gratings (mean luminance of 30 cd/m^2^) were presented to the amblyopic eye, and patches with variable luminance and fixed contrast were presented to the fellow eye. Participants used the method of adjustment to match the perceived luminance (“brightness”) of the gratings across a minimum of four trials. The step size for each button press was a factor of 1.4 (maximum luminance was 60 cd/m^2^). Stimuli were presented in a dimly-lit room using a Cambridge VSG 2/5 graphics card and a Compaq p1210 CRT monitor. Dichoptic stimulus presentation was achieved using a custom-designed 8-mirror stereoscope. Stimulus parameters differed between the psychophysical and fMRI measurements. This was due to differences in the display systems (projectors vs. a CRT monitor) and modifications made to the fMRI stimuli to increase the extent of cortical activation for ROI definition (larger, higher contrast stimuli). To test whether the psychophysical measurements transferred to the scanner environment, participants were asked to describe their perception when viewing the dichoptic fMRI stimulus. Perceptual report of only the fellow eye gratings indicated full suppression of the amblyopic eye. Perceptual report of the amblyopic eye gratings appearing dimmer indicated partial suppression. The results of these tests in the scanner were in agreement with the psychophysical measurements.

### 2.4. MRI

Scanning protocol: Standard monocular retinotopic mapping was conducted in exactly the same way as previously described using wedge and ring stimuli [40,41]. Anatomical and interocular suppression functional images were collected using a Siemens Magnetom 3T scanner located within the Montreal Neurological Institute equipped with an 8-channel coil. T1-weighted anatomical MRI images were collected prior to the functional scans (MPRAGE, 1 × 1 × 1 mm voxels, 2300-ms TR). Functional MRI data were collected from 15 coronal slices oriented to cover the calcarine sulcus and surrounding tissue (in plane resolution 1 × 1 mm, slice thickness 1 mm, 0 mm gap, 1000-ms TR, 30 ms TE).

**Figure 2:**
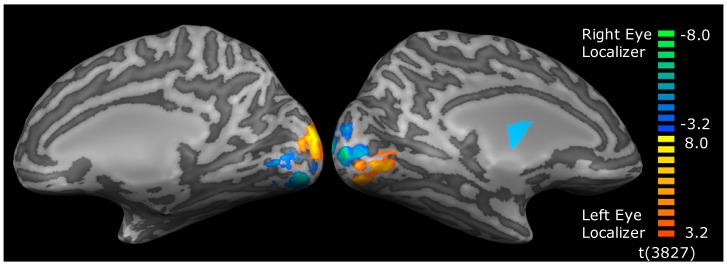
An example of the contrast between the left and right eye ROI localization stimuli in an individual participant. ROIs were generated directly from these activation maps. Eye-specific activation occurred in V1, V2 and V3 for all participants.

**Figure 3:**
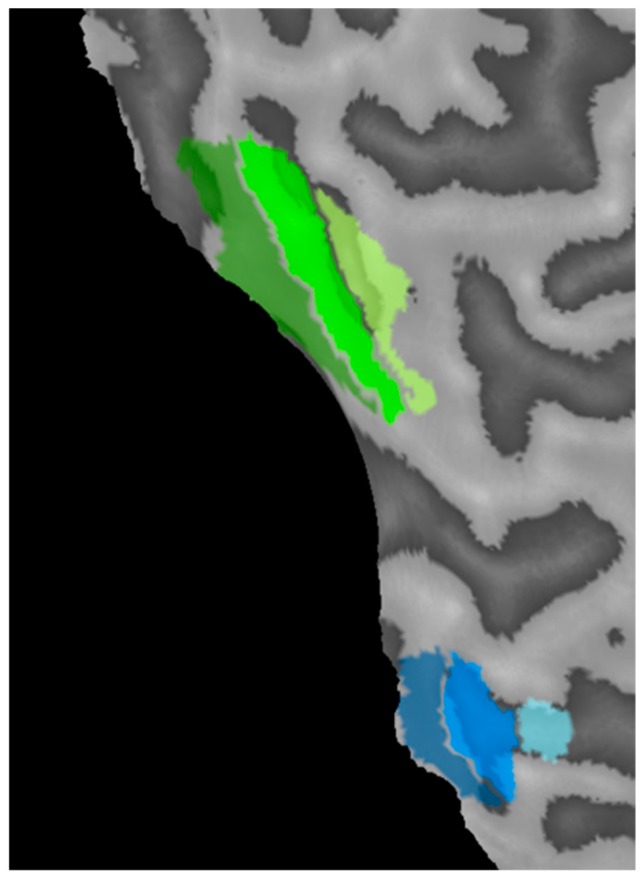
An example of regions of interest derived from the localization stimuli projected onto a region of flattened cortex (the left edge is the calcarine sulcus cut along the vertical meridian). V1, V2 and V3 regions for the amblyopic eye (green) and fellow eye (blue) are shown with V1 closest and V3 furthest from the calcarine sulcus.

Region of interest (ROI) definition: Data were analyzed using BrainVoyager QX. ROIs were defined on a participant by participant basis using the localization stimuli (Figure 1, Columns 1 and 2). This allowed definition of the two retinotopically-distinct regions of cortex that were activated by each eye within retinotopic areas V1, V2 and V3 (Figure 2 and Figure 3). Within V1 for example, for each eye, the lower temporal visual field patch activated a region on and around the upper bank of the calcarine sulcus in the contralateral hemisphere, whereas the upper nasal visual field patch activated a region on and around the lower bank of the calcarine sulcus in the ipsilateral hemisphere. Regions of interest for each eye were defined by calculating a contrast between the two eyes for the two localization stimuli (FDR corrected at *q* < 0.05) and identifying the clusters of voxels driven by each eye in each retinotopic area. Once the clusters had been identified, contiguous, significant voxels that lay within a cubic cm of the peak responding voxel for the cluster were selected for the ROI. 

### 2.5. Analysis

Average time series data were extracted from each ROI for each stimulus condition for each participant. % BOLD change values were calculated by normalizing each point in the time series for each condition to the final 2 TRs of the immediately preceding fixation block. The average % BOLD change within a 16-s window beginning 5 s after stimulus onset was then calculated for each condition for each participant. Prior to analysis, data were averaged across the left and right hemifield ROIs for each participant for each visual area. This was because initial analyses identified no differences between hemifields.

To explore the effects of suppression, comparisons were made between the three stimulus conditions that involved the presentation of all four quadrants simultaneously: two monocular conditions (one for each eye) and one dichoptic condition. Suppression was quantified for each eye separately by comparing the BOLD response within the eye-specific ROIs to the relevant monocular four-quadrant condition (Figure 1, Column 4 or 5) and the dichoptic condition (Figure 1, Column 3). The localization stimuli (Figure 1, Columns 1 and 2) were used for ROI definition only.

Data were analyzed using separate 3-way mixed ANOVA models for each visual area with factors of eye (dominant eye ROI vs. non-dominant eye ROI), condition (dichoptic vs. monocular) and group (amblyopia vs. controls). Post-hoc analyses were conducted using *t*-tests where indicated. The alpha level of statistical testing was set at 0.05 with no correction for multiple comparisons.

To test for an effect of suppression on time to peak of the hemodynamic response function (HRF) within V1, we modelled the HRF for each condition for each participant using the sum of two gamma functions with 6 free parameters. This model provides an accurate and repeatable estimation of time to peak for HRFs generated by block designs [42]. Because a number of HRFs had two distinct peaks, the time to first peak was used for statistical analysis using the ANOVA procedure described above. The first peak was identified using the findpeaks function in the MATLAB signal processing toolbox.

We also examined the feedforward and feedback connections between (1) V1 and V2 and (2) V2 and V3 using an effective connectivity analysis technique applied to the BOLD time course data. The analysis technique utilizes a nonlinear autoregressive exogenous model combined with a least squares statistical method to generate an F-value for each connection for each participant that reflects the strength of connectivity between brain areas within a network (see [36,43] for a detailed description). The F-values were then subjected to standard statistical analysis. Here, we assessed feedforward and feedback connectivity between (1) V1 and V2 and (2) V2 and V3 for each participant for each condition. Connectivity F-values were analyzed using the same ANOVA procedure that was applied to the % BOLD change and time to peak datasets. 

Finally, we conducted a classification analysis to assess whether long-range suppression altered the pattern of activity within the amblyopic eye ROIs (averaged across time points) in a way that was not evident within the % BOLD change or time to peak analyses. Specifically, for each combination of visual area and ROI, we trained classifiers (support vector machine, using the MATLAB function fitcsvm with a linear kernel) to discriminate dichoptic from monocular stimulus presentation. As with the other analyses, the stimuli presented to the eye-specific RIOs were identical in the two conditions. Therefore, above chance classification could only occur if the pattern within the ROIs was differentially affected by presenting the remaining stimulus quadrants dichoptically vs. monocularly. We repeated the classification in a leave-one-out train-and-test procedure, in each case removing a pair of trials (one from each category) from the training set and then testing classification accuracy on the held-out data. For all analyses, we expressed average classifier accuracy in d’ (a unit-free measure of sensitivity). Chance classification performance produces a d’ value of 0. Our hypothesis was that there would be a greater change in the pattern of activity in amblyopic eye ROIs between the dichoptic and monocular conditions than for the fellow eye and control group ROIs. This was because perceptual experience differed substantially between these conditions for the quadrants presented to amblyopic eyes due to long-range suppression. Therefore, we predicted that classification accuracy would be higher for the amblyopic eye ROIs than any of the other ROIs. d’ data were analyzed using a 3-way mixed ANOVA model with factors of eye (dominant eye ROI vs. non-dominant eye ROI), visual area (V1, V2 and V3) and group (amblyopia vs. controls).

## 3. Results

### 3.1. Psychophysical Luminance Matching

Control participants were all able to see all four quadrants within the dichoptic stimulus configuration, and there were no systematic luminance mismatches between the two eyes. Within the amblyopia group, three participants were unable to perceive the quadrants presented to their amblyopic eye, even when interocular luminance was modulated in favor of the amblyopic eye (full suppression: participants RD, GN and JG). Participant AR had an interocular mismatch of 11.9 cd/m^2^, whereby the quadrants shown to the amblyopic eye were seen at a lower luminance than those shown to the fellow eye. Participant AS had a mismatch of 8.1 cd/m^2^.

### 3.2. % BOLD change

**Figure 4:**
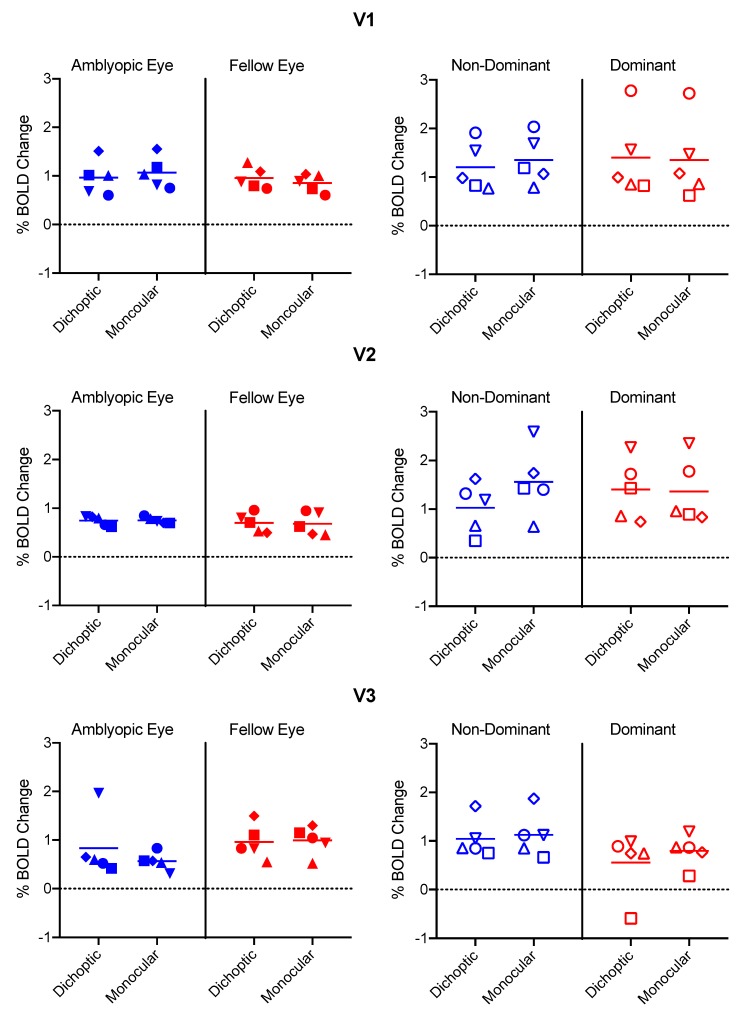
% BOLD change values for each eye and each condition in the amblyopia group (left column) and the control group (right column). Color coding indicates amblyopic/non-dominant eye (blue) and fellow/dominant eye (red). Symbol shapes represent individual participants. The participants with amblyopia who completely suppressed their amblyopic eyes during the dichoptic condition are shown with inverted triangles (RD), circles (GN) and squares (JG).

Fixation task performance means (±SD) were 88.3 ± 9.5% correct for the control group and 90.0 ± 14% correct for the amblyopia group. In area V1, there was a significant interaction between eye and condition (*F_1,8_* = 12.6, *p* = 0.008, *ηp^2^* = 0.61), whereby the BOLD response was lower for the dichoptic than the monocular condition for the amblyopic/non-dominant eye, with the opposite trend apparent for the fellow/dominant eye (Figure 4). This effect was the same for both the amblyopia and control groups (no three-way interaction between eye, condition and group, *p* = 0.8, *ηp^2^* = 0.007). No other interactions or main effects were significant (*p* ≥ 0.05). 

In area V2, there were no significant interactions (*p* > 0.05). There was a significant main effect of group (*F_1,8_* = 6.3, *p* = 0.04, *ηp^2^* = 0.44), whereby the BOLD response was larger for the control than the amblyopia group. No other main effects were significant (*p* > 0.05). 

In area V3, there were no significant main effects or interactions (*p* ≥ 0.05). One participant in the control group exhibited a negative BOLD response within the V3 dominant eye ROI for the dichoptic condition.

**Figure 5:**
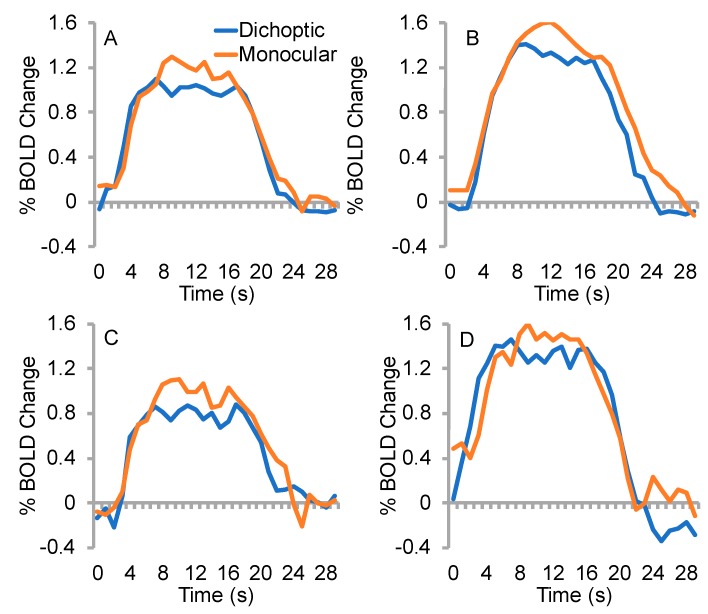
Mean raw % BOLD change time course data for amblyopic/non-dominant eye regions of interest. (**A**) The amblyopia group (*n* = 5), (**B**) the control group (*n* = 5), (**C**) members of the amblyopia group who completely suppressed the amblyopic eye (*n* = 3) or (**D**) exhibited partial suppression (*n* = 2). Between subject variability was substantial (see Figure 4, Row 1; error bars are not shown on time series plots for clarity).

An examination of individual data (Figure 4) indicated that the data for the three participants with amblyopia who perceptually completely suppressed the stimuli presented to their amblyopic eye during dichoptic viewing (data point shapes: inverted triangle, circle, square) typically fell within the range of data from the remaining participants with amblyopia and controls. In fact, the completely suppressed quadrants generated surprisingly robust activity within V1. Figure 5 shows mean raw % BOLD change time courses for the dichoptic and monocular stimulus configurations during amblyopic eye viewing for the amblyopia group as a whole along with means for the sub-group with complete suppression and those with partial suppression. Data for the non-dominant eye of controls are also shown. The members of the amblyopia group with complete suppression exhibited slightly attenuated BOLD responses for both conditions compared to the other groups (Figure 5C). This effect was due to lower overall BOLD responses for participants RD (inverted triangle) and GN (circle) (Figure 4, top left panel).

### 3.3. Time to Peak

**Figure 6:**
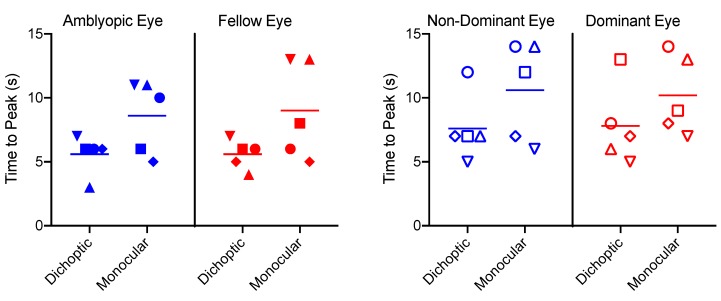
Time to peak data for each participant in the amblyopia group (left panel) and the control group (right panel). Horizontal lines indicate means. Symbols correspond to individual participants in the same way as Figure 4.

**Figure 7:**
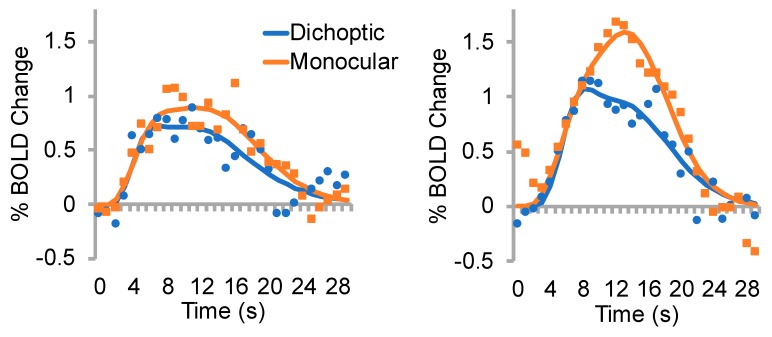
Example datasets for the time to peak analysis. Left panel, amblyopic eye of participant RD; corresponds to filled inverted triangles in Figure 6. Right panel, non-dominant eye of a control participant; corresponds to open inverted triangles in Figure 6. Raw data are shown as points, function fits as solid lines. Data from amblyopic eyes tended to be nosier than data from non-dominant control eyes.

Time to peak data for each eye and condition are shown in Figure 6. Representative individual data are depicted in Figure 7. The time to peak analysis revealed only a significant main effect of condition (*F_1,8_* = 8.4, *p* = 0.02, *ηp^2^* = 0.51), whereby the dichoptic condition had a significantly faster time to peak than the monocular condition. This likely reflects the smaller amplitude of the dichoptic BOLD response (Figure 5). There were no significant main effects or interactions involving the factor of group (*p* > 0.05).

### 3.4. Effective Connectivity 

The feedforward V1 to V2 connectivity analysis revealed only an interaction between eye and group (*F_1,8_* = 11.8, *p* = 0.009, *ηp^2^* = 0.60), whereby the amblyopic eye ROIs exhibited a non-significant (*t_4_* = 2.5, *p* > 0.05, *d* = 1.1) trend for higher connectivity strength than the fellow eye ROIs, while the connectivity strength was relatively equal between the two eyes of controls. No significant main effects or interactions were observed for the V2 to V1 feedback connectivity data. The V2 to V3 connectivity analysis indicated a significant main effect of condition (*F_1,8_* = 8.1, *p* = 0.02, *ηp^2^* = 0.50), whereby connectivity was higher for the monocular condition than the dichoptic condition. In addition, there was an interaction between condition and group (*F_1,8_* = 7.7, *p* = 0.02, *ηp^2^* = 0.49) that appeared to be driven by a significant (*t_4_* = 3.8, *p* = 0.02, *d* = 1.3) increase in connectivity strength for the monocular over the dichoptic condition for the control group that was not present for the amblyopia group. No statistically-significant effects were observed for the V3 to V2 feedback connection.

### 3.5. Classification Analyses

**Figure 8:**
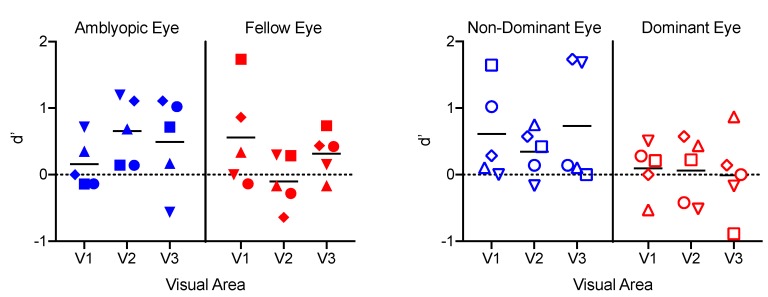
d′ values for each ROI for each participant from a classifier trained to discriminate monocular from dichoptic stimulus presentation.

Classification performance for discriminating dichoptic from monocular stimuli (Figure 8) was above chance for particular combinations of ROI and visual area in the subset of participants within the amblyopia and control groups (Figure 8). This indicates that, in some cases, there were reliable differences in the pattern of BOLD responses, even though the stimuli were identical within the part of the visual field to which each ROI responded. A mixed three-way ANOVA revealed a significant main effect of eye (*F_1,8_* = 7.3, *p* = 0.03, *ηp^2^* = 0.49), whereby the non-dominant eye ROIs exhibited greater classification accuracy than the dominant eyes (*t_9_* = 2.6, *p* = 0.03, *d* = 0.82). There were no other significant main effects or interactions. 

## 4. Discussion

Contrary to our hypothesis, perceptually-suppressed dichoptic stimuli induced robust responses in amblyopic eye ROIs within V1, V2 and V3. These responses did not differ in magnitude or time to peak from those induced in fellow eye ROIs or in controls. In addition, perceptual suppression did not alter effective connectivity between V1, V2 or V3, and a classifier analysis trained to discriminate monocular and dichoptic stimuli for each set of ROIs did not reveal between group differences. Although dichoptic stimuli induced marginally weaker V1 responses than monocular stimuli for amblyopic eye ROIs, this effect also occurred in the non-dominant eye of controls and is likely to be accounted for by normal interocular inhibition [23]. Furthermore, and perhaps most surprisingly, % BOLD change and time to peak data did not differ between participants who completely suppressed the stimuli presented to their amblyopic eyes, participants with partial amblyopic eye suppression and the non-dominant eye of controls. This lack of association between the BOLD data and perception suggests that we were unable to detect long-range interocular suppression in strabismic amblyopia within V1, V2 and V3 using the specific paradigm that we employed. There are at least three possible explanations for this result.

Firstly, this experiment may have been underpowered to detect small differences between viewing conditions and groups. The complexity and duration of the study protocol resulted in low participant enrolment and a sample size that was marginal for formal statistical analysis. However, when one considers that three of the amblyopia group participants perceptually completely suppressed the stimuli presented to their amblyopic eye, the lack of difference between groups is striking. 

Secondly, long-range interocular suppression may alter neural activity in ways that were not detectable by our fMRI protocol. For example, our fMRI measurements would be insensitive to sub-second changes in the relative timing of neural responses to each eye. Timing differences between the two eyes have been reported in strabismic amblyopia [44], and it is conceivable that a relatively delayed neural response to the amblyopic eye could contribute to perceptual suppression under dichoptic viewing conditions. Further studies using techniques with a high temporal resolution such as magnetoencephalography will be required to address this possibility. In addition, recent primate neurophysiology data [21] indicate that interocular suppression in amblyopia is associated with a weak excitatory drive from the amblyopic eye rather than abnormally strong suppression of the amblyopic eye inputs to primary visual cortex. Our fMRI protocol was not designed to detect an overall attenuation of activity in response to the amblyopic eye because the data were expressed as a percent change from the baseline. Therefore, is it possible that weak excitatory signals from the amblyopic eye in early visual cortex underlie long-range suppression. 

Finally, although weak dichoptic masking is evident in single cell recordings within V1 for non-overlapping stimuli in close proximity [27], long-range suppression may involve brain areas outside of V1, V2 and V3 that have large visual receptive fields. In this experiment, the fMRI protocol involved a small number of tightly-packed slices centered on V1 to optimize the spatial and temporal resolution. Therefore, data were not collected for higher level visual areas. Evidence that higher level areas may be involved in interocular suppression includes the recent observation that adults with strabismic amblyopia exhibit a shift in visual attention away from the amblyopic eye when both eyes are open [45]. This psychophysical effect may involve the intraparietal sulcus and superior parietal lobe, both of which are associated with attentive tracking [46]. If abnormal interocular allocation of attention does play a role in long-range suppression, it is likely that the brain areas involved fall outside of the region of cortex that was imaged in this experiment.

Our results are consistent with the only previous fMRI study that investigated long-range suppression in strabismic amblyopia. Chen et al. [47] presented counterphase checkerboard stimuli to the upper visual field of the fellow eye and recorded the V1 BOLD response for stimuli presented to the lower visual field of the deviated eye. Reduced V1 activation induced by long-range suppression was found in the subset of participants with strabismus, but no amblyopia; however, no evidence of suppression was found in the V1 BOLD response for participants with strabismic amblyopia. In addition, an EEG study that used frequency tagging to assess neural responses to each eye under dichoptic viewing conditions observed reduced dichoptic masking in participants with strabismic or mixed amblyopia relative to controls [48]. Finally, although perceived luminance is affected by long-range suppression in strabismic amblyopia [28], suprathreshold contrast perception appears to be relatively unaffected [28,49]. Together, these results suggest that interocular suppression in strabismic amblyopia cannot easily be explained by an early, active suppression of the neural response to the suppressed eye.

## 5. Conclusions 

We did not observe evidence for long-range interocular suppression at V1, V2 or V3 within our sample of adults with strabismic amblyopia. This was the case even when amblyopic eye stimuli were completely suppressed from perception. We note that our sample was small and subtle group differences may have emerged with more participants. A larger study is required to replicate these initial findings. However, based on the available data, we speculate that interocular timing differences and/or higher level brain areas may underpin long-range suppression in strabismic amblyopia.

## Figures and Tables

**Table 1:** Characteristics of participants in the amblyopic group. The data point column refers to the symbols used to depict individual participant data in Figures 4, 6 and 8.

Participant	Age	Type	Refraction	Acuity	Deviation	History, Stereo	Data Point
AR	47/M	RE LE strab	Ø Ø	20/20 20/50	XT 1°	Detected age 6 y, no patching, no surgery, no stereopsis	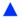
RD	49/M	RE LE strab	+3.00 DS +4.00 DS	20/15 20/125	ET 1°	Detected age 6 y, glasses 6 y, no other treatment, local stereopsis (200 arcsec)	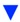
GN	30/M	RE mixed LE	+5.00–0.00 × 120° +3.50–1.00 × 75°	20/70 20/20	ET 8°	Detected age 2 y, strabismus surgery ages 2–6 y, no stereopsis	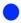
JG	21/M	RE LE strab	−2.00–0.50 × 150 −1.00 DS	20/20 20/100	ET 3°	Detected age 5 y, patching for 3 y, no surgery, local stereopsis 200 arcsec	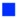
AS	21/F	RE LE strab	Ø −0.5 DS	20/160 20/20	ET 15°	Detected age 4 y, patching at 4 y for 6 m, surgery at 7 y, no stereopsis	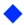
